# Postexercise essential amino acid supplementation amplifies skeletal muscle satellite cell proliferation in older men 24 hours postexercise

**DOI:** 10.14814/phy2.13269

**Published:** 2017-06-08

**Authors:** Paul T. Reidy, Christopher S. Fry, Jared M. Dickinson, Micah J. Drummond, Blake B. Rasmussen

**Affiliations:** ^1^ Center for RecoveryPhysical Activity and NutritionUniversity of Texas Medical BranchGalvestonTexas; ^2^ Department of Nutrition and MetabolismUniversity of Texas Medical BranchGalvestonTexas

**Keywords:** Stem cell activation, strength training

## Abstract

Aged skeletal muscle has an attenuated and delayed ability to proliferate satellite cells in response to resistance exercise. The mechanistic target of rapamycin complex 1 (mTORC1) signaling pathway is a focal point for cell growth, however, the effect of postexercise mTORC1 activation on human skeletal muscle satellite cell (SC) proliferation is unknown. To test the proliferative capacity of skeletal muscle SC in aging muscle to a potent mTORC1 activator (i.e., EAA; essential amino acids) we recruited older (~72y) men to conduct leg resistance exercise (8setsx10reps) without (−EAA;* n* = 8) and with (+EAA:* n* = 11) ingestion of 10 g of EAA 1 h postexercise. Muscle biopsies were taken before exercise (Pre) and 24 h postexercise (Post) for assessment of expression and fiber type‐specific Pax7^+^ SC, Ki67^+^Pax7^+^ SC and MyoD^+^ SC. −EAA did not show an increase in Pax7^+^ satellite cells at Post(*P* > 0.82). Although statistical significance for an increase in Pax7 +  SC at 24 h post‐RE was not observed in +EAA versus −EAA, we observed trends for a treatment difference (*P* < 0.1). When examining the change from Pre to Post trends were demonstrated (#/myofiber: *P* = 0.076; and %/myonuclei: *P* = 0.065) for a greater increase in +EAA versus −EAA. Notably, we found an increase SC proliferation in +EAA, but not −EAA with increase in Ki67^+^ SC and MyoD^+^ cells (*P* < 0.05). Ki67^+^ SC also exhibited a significant group difference Post (*P* < 0.010). Pax7^+^ SC in fast twitch myofibers did not change and were not different between groups (*P* > 0.10). CDK2, MEF2C, RB1 mRNA only increased in +EAA (*P* < 0.05). Acute muscle satellite cell proliferative capacity may be partially rescued with postexercise EAA ingestion in older men.

## Introduction

The loss of skeletal muscle mass (Lexell et al. 1988; Doherty 2003) and quality (Koopman and van Loon 2009) is common with aging. This loss highlights the development of sarcopenia, where diminished muscle mass and strength are major contributors engendering loss of independence and quality of life for older adults (Cruz‐Jentoft et al. 2010). Current research suggests that reductions in the ability to stimulate muscle protein synthesis (Koopman and van Loon 2009; Dickinson et al. 2013) and promote proliferation and differentiation of muscle satellite cells (Verdijk et al. 2014) may be important contributors to the development of sarcopenia. In addition, muscle satellite cells may also play an important role in the maintenance of muscle quality (Fry et al. 2014a), which is especially relevant during aging (Fry et al. 2015).

It is well known that exercise and the ingestion of essential amino acids, in particular the amino acid leucine, are important stimulators of muscle protein synthesis through activation of the mechanistic target of rapamycin complex 1 (mTORC1) signaling pathway (Walker et al. 2011). While an anabolic resistance to the independent effects of exercise and EAA or protein is prevalent with aging (Dickinson et al. 2013), combining the two stimuli shows promise in combating sarcopenia (Walker et al. 2011; Dickinson et al. 2013) via the ability for this combination to maximally stimulate mTORC1 and upregulate the translation initiation machinery. Indeed, we have recently demonstrated that provision of leucine‐enriched EAA mixture following a bout of high‐intensity resistance exercise (RE) stimulates mTORC1 and prolongs myofibrillar protein synthesis for up to 24 h post‐RE in the very same cohort of older men we examined in this study (Dickinson et al. 2014) whereas, in the absence of EAA this mTORC1 response is blunted in older adults (Fry et al. 2011).

As a result of the amplified level of translation in response to each acute bout of RE (Walker et al. 2011; Dickinson et al. 2013), myofiber growth is thought to occur as a result of repeated exposures to a RE stimulus (Blaauw and Reggiani 2014; Brook et al. 2015). Concurrent with this response, transcriptional regulation (Chen et al. 2002), myogenic proliferation and differentiation occur in dormant satellite cells (SC). A prevailing theory is that these dormant satellite cells become active and are fused as nuclei to myofibers to meet the demands of the enlarged myofiber. Although satellite cells may not be necessary for hypertrophy to occur (Blaauw and Reggiani 2014), it is possible that they modulate the magnitude of muscle hypertrophy (Petrella et al. 2008; Bellamy et al. 2014) or also directly influence areas of muscle maintenance and quality (Blaauw and Reggiani 2014; Fry et al. 2014a, 2015). A host of evidence has suggested increased SC activation and content following RE in human skeletal muscle (Blaauw and Reggiani 2014), yet we and others have demonstrated a blunting of (Walker et al. 2012) or a delayed ability (Snijders et al. 2014) to activate and increase the SC pool in older men compared with a younger cohort. EAA and leucine provision has been shown to upregulate SC activity via mTORC1 (Kornasio et al. 2009; Alway et al. 2013; Rodgers et al. 2014), which is also a regulatory point of myogenic differentiation (Jash et al. 2014). Also, provision of whey protein has been shown to augment postexercise CDK2 mRNA expression with younger (Hulmi et al. 2009) and older men (Hulmi et al. 2008). However, the promising combined effects of RE and postexercise EAA supplementation on SC activity and content have not been examined in older adult humans.

Therefore, we hypothesized that EAA ingestion, which we have previously shown to potently activate mTORC1 (Dickinson et al. 2014) following an acute bout of leg resistance exercise, would enhance skeletal muscle satellite cell proliferative capacity and content in older men.

## Methods

### Participants

To address our current hypothesis, we analyzed samples from 19 healthy older men that were collected in two of our previous studies that used identical study designs (Walker et al. 2012; Dickinson et al. 2014) (Table 1). The two previous studies were part of a larger set of related studies that used identical study designs (Walker et al. 2012; Dickinson et al. 2014). All participants were considered healthy enough to conduct high‐intensity RE. Screening was performed with clinical history, physical examination, and laboratory tests, including complete blood count with differential, liver and kidney function tests, coagulation profile, fasting blood glucose, oral glucose tolerance test, hepatitis B and C screening, HIV testing, thyroid‐stimulating hormone, urinalysis, and drug screening. All participants gave informed written consent prior to participation, which was approved by the Institutional Review Board of the University of Texas Medical Branch (which is in compliance with the Declaration of Helsinki as revised in 1983).

**Table 1:** Participant characteristics

	Age (y)	Height (cm)	Weight (kg)	BMI (kg/m^2^)	FFM (kg)	% Body fat	MFA (*μ* ^2^)
+EAA	72.5 ± 2.2	175.2 ± 1.5	80.8 ± 2.6	26.3 ± 0.7	55.3 ± 1.7	28.9 ± 1.7	4960 ± 470
−EAA	70.0 ± 2.2	172.9 ± 2.9	76.2 ± 3.4	25.4 ± 0.6	53.3 ± 2.2	25.8 ± 1.8	3802 ± 370

Values are means ± SE. Mean myofiber area (MFA). All *P* > 0.10, except for body weight.

Maximal knee extensor muscle strength was determined for each participant on two separate occasions (separated by approximately 7 days) using a one‐repetition maximum (1RM) performed on a leg extension device (Cybex‐VR2, Medway, MA). The first 1RM measurement was obtained during the initial screening visit and the second 1RM measurement was obtained approximately 1 week prior to study participation. The highest weight lifted between the two measurements was considered the participant's 1RM.

### Experimental Design

Participants were placed into one of two experimental groups, no essential amino acids (−EAA), or essential amino acids (+EAA). All groups completed a two‐day experimental trial (over two consecutive days), which was identical for each group with the exception of a 10 g EAA beverage that was ingested following exercise (+EAA). Participants were housed in the Institute for Translational Sciences Clinical Research Center (ITS‐CRC) of the University of Texas Medical Branch for the entirety of the two‐day experimental trial. Only nine of the +EAA and five of the −EAA subjects had suitable muscle cross‐sections for our primary analysis.

The evening prior to the experimental study, subjects were admitted to the ITS‐CRC as previously reported (Walker et al. 2012; Dickinson et al. 2014). Subjects were studied in the fasted state, under basal conditions. The first morning of the experimental trial an 18‐gauge polyethylene catheter was inserted retrograde in a hand vein of the contra‐lateral arm, which was kept in a heated pad for arterialized blood sampling. The first muscle biopsy was obtained from the lateral portion of the vastus lateralis of the leg with the biopsy site between 15 and 25 cm from the mid patella. The biopsy was performed using a 5 mm Bergström biopsy needle (Bergstrom 1962) with suction under sterile procedure and local anesthesia (1% lidocaine). Following the muscle biopsy, participants were seated on the leg extension device to begin the exercise portion of the study. Participants completed 8 sets of 10 repetitions at an intensity targeted to require 60–70% 1RM with 3 min of rest between each set. Total time for the exercise period was approximately 30 min. At 1 h postexercise, participants in the +EAA group ingested 10 g of EAA in composition that has been previously described (Glynn et al. 2010). Participants in the −EAA group did not ingest nutrients postexercise until the standardized evening meal on the first day of the experimental trial, ~1700 h. Blood was obtained at 2 h postexercise period to assess amino acid concentrations using our standard methods. However, a few participants had issues with the catheter at this time so we do not have AA data for them (only for +EAA, *N* = 10; −EAA, *N* = 7). In the +EAA, but not −EAA, a muscle biopsy was obtained 1 h following EAA ingestion (2 h postexercise) for assessment of skeletal muscle mRNA expression. Participants remained in the ITS‐CRC and were encouraged to move around the unit to compile between 1000 and 1500 steps (to avoid prolonged bed rest), which was monitored by a study nurse. Participants were fed the identical dinner and snack from the previous night prior to an overnight fast in preparation for the second day of the experimental trial. On the second morning of the experimental trial, another catheter was inserted for arterialized blood sampling. A muscle biopsy was sampled at 24 h postexercise from a new incision. Next, participants were given a meal and discharged from the unit. Muscle tissue from all biopsies was immediately blotted and frozen in liquid nitrogen and stored at −80°C until analysis. Approximately 20 mg of sample was carefully embedded in Tissue Tek optimal cutting temperature (OCT; Thermo Fisher Scientific, Rockford, IL) on a cork and frozen in liquid nitrogen cooled isopentane for histochemical analysis.

### Essential amino acid solution composition

Four +EAA participants were given the following amounts of each EAA in a 10 g mixture; histidine (1.1 g), lysine (1.55 g), methononine (0.3 g), threonine (1.45 g), phenylalanine (1.55 g), valine (1.2 g), isoleucine (1 g) and leucine (1.85 g). The remaining seven +EAA participants were given the following amounts of each EAA in a 10 g mixture; histidine (0.8 g), lysine (1.2 g), methononine (0.3 g), threonine (1.0 g), phenylalanine (1.4 g), valine (1.0 g), isoleucine (0.8 g) and leucine (3.5 g). The effect of EAA composition was tested on our outcomes (IHC or mRNA), however, no differences were observed for any outcome and thus subjects that ingested EAA were grouped. Also, both EAA groups activated mTORC1 signaling (Dickinson et al. 2014) above that which we previously demonstrated for the EAA‐ group (Fry et al. 2011). EAA were dissolved in a noncaloric and caffeine‐free flavored beverage (350 mL) to increase palatability.

### RNA Extraction and semiquantitative real‐time PCR

RNA isolation was performed as we have previously described (Drummond et al. 2009; Fry et al. 2011). Total RNA was isolated by ~30 sec of homogenizing 10–20 mg tissue with a hand‐held homogenizing dispenser (T10 Basic Ultra Turrax, IKA, Wilmington, NC) in 1 mL of Tri reagent. The RNA was separated into an aqueous phase using 0.2 mL of chloroform and subsequently precipitated from the aqueous phase using 0.5 mL of isopropanol. RNA was washed twice with 1 mL of 75% ethanol, air‐dried, and suspended in a known amount of nuclease‐free water. RNA concentration was determined using a NanoDrop 2000 spectrophotometer (Thermo Fisher Scientific, Wilmington, DE). RNA was again isolated on available samples with 475*μ*L of aqueous phase extracted.

A total of 2 *μ*g of RNA was reverse transcribed into cDNA according to the directions provided by the manufacturer (iScript, BioRad, Hercules, CA). Real‐time qPCR was carried out using the PrimePCR system with a CFX Connect PCR cycler (BioRad). cDNA was analyzed with SYBR green fluorescence (iQ SYBR green supermix; BioRad). Validated gene targets and unique Bio‐Rad assay ID's for the following targets (PAX7, MyoD, MyoG, mib2, MEF2C, CDK6, CCND1, RB1, NPM1, and MDM2) are highlighted in Table S1. These genes were chosen for expression analysis due to their involvement in satellite cell specification, activation, and differentiation. Additional target genes encode for proteins involved in cell cycle progression and are integral for satellite cell activation and proliferation. Standard practice in our laboratory has repeatedly demonstrated that B2M (*β*₂ microglobulin) is stable during acute exercise/nutritional interventions, including this investigation, thus it was used as a normalization/housekeeping gene. Relative fold changes were determined from the Ct values using the 2^−ΔΔCt^ method (Livak and Schmittgen 2001).

### Immunohistochemistry

Immunohistochemical techniques were conducted as previously described (Fry et al. 2014b, 2016). Samples were removed from cork at −25°C in a cryostat (Thermo HM525‐NX) where serial sections were cut (7 *μ*m). Pre and post samples for the same subject were placed on the same Fisherbrand Superfrost^®^/Plus microscope slides (Fisher Scientific) for analysis of; (1) fiber‐type specific satellite cell (Pax7^+^) content and myonuceli, (2) double stain of proliferative (Ki67^+^) satellite cells (Pax7^+^) and (3) proliferative MyoD+ cells. Following cutting, hydrophobic marker was used to separate the sections, dried at room temperature and then stored at −20^°^C until analysis. Only 9 of the +EAA and 5 of the −EAA subjects had suitable muscle cross‐sections for analysis.

#### Fiber‐type specific satellite cells (Pax7^+^)

Methods have been published previously (Fry et al. 2014b, 2016). Briefly, Sections were fixed in ice‐cold acetone then incubated primary antibodies against MHC I (BA.D5 IgG2b, 1:50, Developmental Studies Hybridoma Bank, Iowa City, IA) and Laminin (L9393, 1:200, Sigma‐Aldrich, St. Louis, MO). On day 2 sections were incubated in Alexa Fluor 647‐conjugated goat anti‐mouse IgG2b (for MHC I: 1:250, Invitrogen, Carlsbad, CA) and Alexa Fluor 594 goat anti‐rat IgG1 (for laminin: 1:500, Invitrogen, Carlsbad, CA), washed and then incubated with a primary antibody against Pax7 (1:100, Developmental Studies Hybridoma Bank, Iowa City, IA). On day 3 sections were incubated with goat anti‐mouse IgG biotin –SP‐conjugated (1:1000) (Jackson Immuno Research, Cat #115‐065‐205), streptavidin‐horseradish peroxidase conjugate and then reacted with the tyramide signal amplification kit (ThermoFisher, Cat #T20932) followed by mounting in DAPI containing mounting media (Vector) and allowed to air dry. The staining protocol resulted in DAPI‐positive nuclei staining blue, Pax7 +  cells (stained yellow), MHC I (stained purple), MHC II (Black‐negative staining) and laminin basement membrane (stained red) of a muscle fiber. Myonuclei were manually counted in images captured with a 100–400× total magnification using AxioVision 4.9.1 software to determine the number of myonuclei per fiber. A nucleus was identified as a myonucleus if it met one of the following criteria: (1) it is clearly located within the laminin boundary; (2) it is on the boundary facing inside the fiber; or (3) greater than 50% of the area falls inside the laminin boundary. Rapid, repeated manual switching back‐and‐forth between single channel laminin images and merged laminin/DAPI images was used to determine the location of a nucleus as inside or outside of the laminin boundary. Following counting of myonuclei within an image, fiber number was quantified manually to express the number of myonuclei per fiber. Pax7 +  nuclei/myofiber, % SC, and myonuclei per fiber was determined from 100 to 200 cross‐sectional muscle fibers at each time point, as Mackey et al. (2009) recommend that counting from a minimum of 125 muscle fibers is needed to obtain reliable data for satellite cell content. Two of the participants in the +EAA group did not have enough MHC II fibers (<50) for analysis. To avoid bias during counting, sections were imaged, coded and then analyzed by a blinded investigator. The mean myofiber area (MFA) at pre was assessed using images from the above stain with analysis with semiautomatic muscle analysis using segmentation of histology: a MATLAB application (SMASH) (Smith and Barton 2014).

#### Double stain of proliferative (Ki67^+^) satellite cells (Pax7^+^)

Sections were fixed for 7 min in 4% PFA and washed in PBS. Following antigen retrieval with Na citrate (10 mmol/L, pH 6.5) at 92°C water bath and a PBS rinse, sections were treated for 7 min in H₂O₂ (3% in PBS) to block endogenous peroxidases. After a 3 × 3 min rinse in PBS, sections were blocked for 1 h in 1% TSA blocking solution (Invitrogen) at RT. Sections were incubated for 1 h at RT and then overnight at 4°C with a primary antibody against rabbit anti‐Ki67 (1:100) (Biocare Medical, cat#CRM325B) and mouse anti‐Pax7 (Concentrate 1:100) from DHSB in 1% TSA at 4°C. On the second day of staining a 3 × 3 min wash in PBS proceeded a 80 min incubation in goat anti‐rabbit IgG AF555 (1:250) (Invitrogen, Cat # A‐21429) and goat anti‐mouse IgG biotin –SP‐conjugated (1:1000) (Jackson Immuno Research, Cat #115‐065‐205). Sections were washed 3 × 3 min in PBS and then exposed to a 1 h incubation of Streptavidin‐horseradish peroxidase conjugate (1:100) in PBS, washed 3 × 3 min in PBS, and incubated for 20 min in Alexa Fluor 488 (1:200, (Invitrogen Cat# T20932)) in amplification diluents. Following a 3 × 3 min wash in PBS, sections were incubated 10 min in DAPI (Invitrogen, cat#D3571); dilute (100 *μ*m) stock (1:10,000 in PBS) at RT. After a quick wash slides were mounted in medium mounting media (Vector) and allowed to air dry prior to imaging. The staining protocol resulted in satellite cells as green, Ki67 as red, and DAPI as blue. Satellite cells undergoing proliferation were identified as Pax7 + , Ki67 + , and DAPI+. Ki67 +  satellite cells were normalized to total satellite cell number (all Pax7 +  cells) and presented as a percentage of total satellite cells.

#### MyoD+ cells

Sections were fixed in ice‐cold acetone for 10 min followed by 3 × 3 min rinse in PBS. Sections were treated for 7 min in H₂O₂ (3% in PBS) to block endogenous peroxidases. After a 3 × 3 min rinse in PBS, sections were blocked for 1 h in 2.5% normal horse serum (Vector) at RT. Sections were incubated for 1 h at RT and then overnight at 4°C with a primary antibody against mouse anti‐MyoD (1:100, BD Biosciences cat #554130). The following day, sections were rinsed 3 × 5 min with PBS before and after 1 h incubation with goat anti‐mouse IgG biotin –SP‐conjugated (1:1000) in 2.5% NHS (Jackson Immuno Research, Cat #115‐065‐205) at RT. Sections were washed in PBS (3 × 3 min) and then exposed to a 1 h incubation of Streptavidin‐horseradish peroxidase conjugate (1:100) in PBS, washed, 3 × 3 min in PBS, and incubated for 20 min in Alexa Fluor 488/594 (1:200) in amplification diluents. Following a 3 × 5 min wash in PBS, sections were mounted in DAPI containing medium mounting media (Vector) and allowed to air dry. The staining protocol resulted in DAPI‐positive nuclei staining blue, laminin staining green, and MyoD+ cells stained red. Activated satellite cells were identified as MyoD+ and DAPI+ residing within the laminin border. MyoD+ satellite cells were normalized to fiber number.

### Statistical analysis

Following tests for variance and normality of the data, independent t‐tests were used to compare participant characteristics between groups. A two‐way ANOVA with repeated measures on the time factor was used to test time (pre vs. post) by group (+EAA vs. −EAA) differences. Additionally, Pax7 data was analyzed as a change from preexercise (delta Pre‐Post) and compared using a student's TTEST. Post hoc testing was performed using Sidak's multiple comparison test. Significance for all analyses was set to *P *≤* *0.05.

## Results

### Subject characteristics

Subject characteristics at baseline were not different (*P* > 0.10) between +EAA and −EAA (Table 1). Similar findings were observed in the subset of participants who had histology data, however, there was a trend (*P* < 0.10) for body weight in +EAA (81.7 ± 3.1 kg) to be greater than with −EAA (71.0 ± 3.5 kg).

### Amino acid concentrations

Blood phenylalanine and leucine increased (*P* < 0.05) in +EAA, but not −EAA, at 2 h postingestion (Table 2). Muscle intracellular phenylalanine and leucine demonstrated the same pattern (data not shown).

**Table 2:** Blood concentrations of phenylalanine and leucine before and after resistance exercise in older men with (+EAA) or without (−EAA) postexercise essential amino acid (EAA) ingestion 1 h postexercise

	Pre	2 h Post	24 h Post
Blood phenylalanine, *μ*mol/l			
+EAA	66 ± 2	176 ± 14^a^	70 ± 3
−EAA	65 ± 4	63 ± 4^b^	69 ± 4
Blood leucine, *μ*mol/l			
+EAA	134 ± 3	534 ± 52^a^	144 ± 8
−EAA	146 ± 9	121 ± 7^b^	148 ± 5

Values are means ± SE.

a Significantly different from Pre (*P* < 0.05),

b Significantly different than +EAA (*P* < 0.05).

### MHC composition

MHC composition did not change (*P* > 0.10) pre to post and was similar between +EAA (46.9 ± 3.3% MHC I) and −EAA (46.7 ± 4.4% MHC I) treatments.

### Muscle mRNA expression

**Figure 1:**
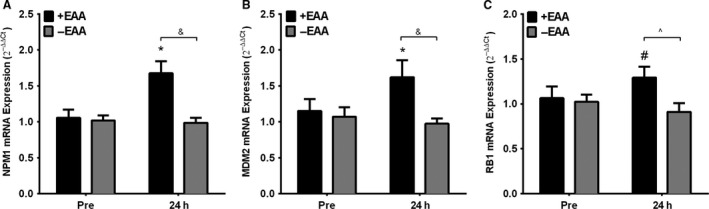
EAA ingestion following resistance exercise influences cell cycle and satellite cell marker gene expression in older men. Skeletal muscle *vastus lateralis* mRNA expression of CDK2 (A), MyoD (B), MEF2C (C) and MyoG (d) in older adult men before (Pre) and 24 h (Post) following a bout of high‐intensity resistance knee extension exercise with (+EAA) or without (−EAA) postexercise EAA ingestion. Data are mean ± SEM. * *P* < 0.05 vs Pre for that group, # *P* < 0.10 versus Pre for that group. Normalized to B2M using the 2^−ΔΔCt^ method.

**Figure 2:**
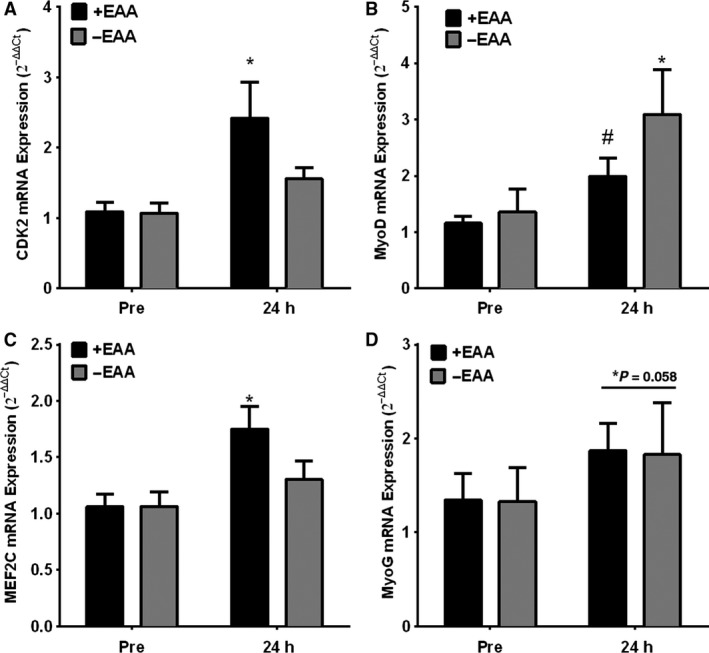
EAA ingestion following resistance exercise influences gene expression in older men. Skeletal muscle *vastus lateralis* mRNA expression of NPM1 (A), MDM2 (B) and RB1 (C) in older adult men before (Pre) and 24 h (Post) following a bout of high‐intensity resistance knee extension exercise with (+EAA) or without (−EAA) post‐exercise EAA ingestion. Data are mean ± SEM. * *P* < 0.05 versus Pre for that group, & *P* < 0.05 treatment effect at Post. # *P* < 0.10 versus Pre for that group, ^ *P* < 0.10 treatment effect at Post. Normalized to B2M using the 2^−ΔΔCt^ method.

CCND1, CDK6, PAX7, and mib2 expression had no main effects of time, treatment or interactions (*P* > 0.10) and did not change pre to post (Table 3). CDK2 demonstrated a significant main effect of time (*P* < 0.01), which was driven by a significant increase following +EAA (*P* = 0.01), but not −EAA treatment (*P* = 0.54). MEF2C demonstrated a significant main effect of time (*P* = 0.02), which was driven by a significant increase following +EAA (*P* = 0.01), but not −EAA treatment (*P* = 0.60). NPM1 demonstrated significant main effects of time, treatment and an interaction (Fig. 1; *P* < 0.02), which was driven by a significant increase following +EAA (*P* < 0.01) but not −EAA treatment (*P* = 0.98) that resulted in a difference between treatments at post (*P* < 0.01). RB1 demonstrated a significant interaction (*P* = 0.03), which was driven by a trend for an increase following +EAA (*P* = 0.06) but not −EAA treatment (*P* = 0.55) that resulted in a difference between treatments at post (*P* = 0.05). MyoD demonstrated a significant main effect of time (Fig. 2; *P* < 0.01), which was driven by an increase following +EAA (*P* = 0.05) and also −EAA treatment (*P* < 0.01). MyoG demonstrated a trend for a main effect of time (*P* = 0.06), which was not a significant increase for following specific +EAA (*P* = 0.24) or −EAA treatment (*P* = 0.38).

**Table 3:** mRNA expression in *vastus lateralis* of before and after (24 h) resistance exercise in older men with (+EAA) or without (−EAA) postexercise essential amino acid (EAA) ingestion 1 h postexercise

	+EAA		−EAA	
	Pre	24 h	Pre	24 h
CCND1	1.13 ± 0.17	1.39 ± 0.28	1.23 ± 0.21	1.30 ± 0.15
CDK6	1.14 ± 0.18	1.16 ± 0.16	1.09 ± 0.16	1.22 ± 0.14
PAX7	1.26 ± 0.21	1.33 ± 0.27	1.19 ± 0.25	0.87 ± 0.13
mib2	1.37 ± 0.29	1.18 ± 0.26	1.23 ± 0.27	1.18 ± 0.14

Values are means ± SE. Normalized to B2M using the 2^−ΔΔCt^ method.

We were able to take a biopsy 1 h post EAA (2 h postexercise) following only the +EAA treatment to capture the effects of EAA ingestion (Table 4). CDK2, CCND1, CDK6, MEF2C, PAX7, MDM2, NPM1, RB1, MyoG, and mib2 expression and did not change (*P* > 0.100) pre to 1 h post‐EAA. However, MyoD increased (*P* < 0.05) pre to 1 h post‐EAA.

**Table 4:** mRNA Expression in *vastus lateralis* before and after amino acid (EAA) ingestion 1 h postingestion (2 h postexercise) in older men

+EAA	Pre	1 h post‐EAA	*P*‐value
CDK2	1.09 ± 0.13	1.32 ± 0.13	0.11
CCND1	1.13 ± 0.17	1.29 ± 0.18	0.36
CDK6	1.14 ± 0.18	1.12 ± 0.17	0.95
MEF2C	1.06 ± 0.11	1.17 ± 0.15	0.54
PAX7	1.26 ± 0.21	0.87 ± 0.13	0.10
MDM2	1.07 ± 0.12	1.22 ± 0.13	0.72
NPM1	1.06 ± 0.11	1.09 ± 0.11	0.56
RB1	1.07 ± 0.13	1.10 ± 0.11	0.71
MyoD	1.16 ± 0.13	1.90 ± 0.27	**0.02**
MyoG	1.35 ± 0.28	1.49 ± 0.43	0.73
mib2	1.37 ± 0.29	1.22 ± 0.23	0.65

Values are means ± SE. Normalized to B2M using the 2^−ΔΔCt^ method.

The *P*‐value for MyoD expression is bolded to denote statistical significance at a *P* < 0.05.

### Characteristics of IHC analysis

There were no differences in the number of fibers counted for analysis of Pax7 +  satellite cells per myofiber for MHC I myofibers or when all myofiber types were pooled (*P* > 0.10). However, there was a trend for a main effect of treatment in MHC II myofibers (*P* = 0.09), but no pairwise differences. There were no differences in the number of fibers counted for analysis of myonuclei per myofiber for MHC I or MHC II myofibers or when all myofiber types were pooled (*P* > 0.10) (Table 5).

**Table 5:** The total number of fibers counted for analysis of satellite cells (SC) and myonuclei (MyoN) before and after resistance exercise in older men with (+EAA) or without (−EAA) postexercise essential amino acid (EAA) ingestion 1 h postexercise

	MHC I		MHC II		All	
	Pre	Post	Pre	Post	Pre	Post
SC # fibers						
+EAA	280 ± 62	263 ± 66	282 ± 32	231 ± 47	563 ± 86	494 ± 91
−EAA	337 ± 59	291 ± 47	432 ± 114^a^	266 ± 50	769 ± 173	557 ± 90
MyoN # fibers						
+EAA	161 ± 13	146 ± 25	171 ± 16	130 ± 29	332 ± 9	276 ± 23
−EAA	149 ± 19	156 ± 14	170 ± 31	154 ± 19	319 ± 44	310 ± 26

Values are means ± SE.

a significantly different than +EAA (*P* < 0.05).

### Muscle myonuclei per fiber

Myonuclei per myofiber for MHC I myofibers, MHC II myofibers and when all myofiber types pooled were not different at pre (*P* > 0.10). There were no main effects of time of interactions (*P* > 0.10), with the exception of a trend for a main effect of treatment when all myofibers where pooled. Yet, exploratory pairwise comparisons could not detect differences at pre or post individually (*P* > 0.10).

### Centrally‐located myonuclei

As a measure of muscle damage due to the exercise bout, the relative frequency of fibers containing a centrally located myonucleus was determined. A main effect of time was found, with an elevation in the frequency of fibers containing centrally located myonuclei (data not shown).

### Muscle Pax7 +  satellite cell content

**Figure 3:**
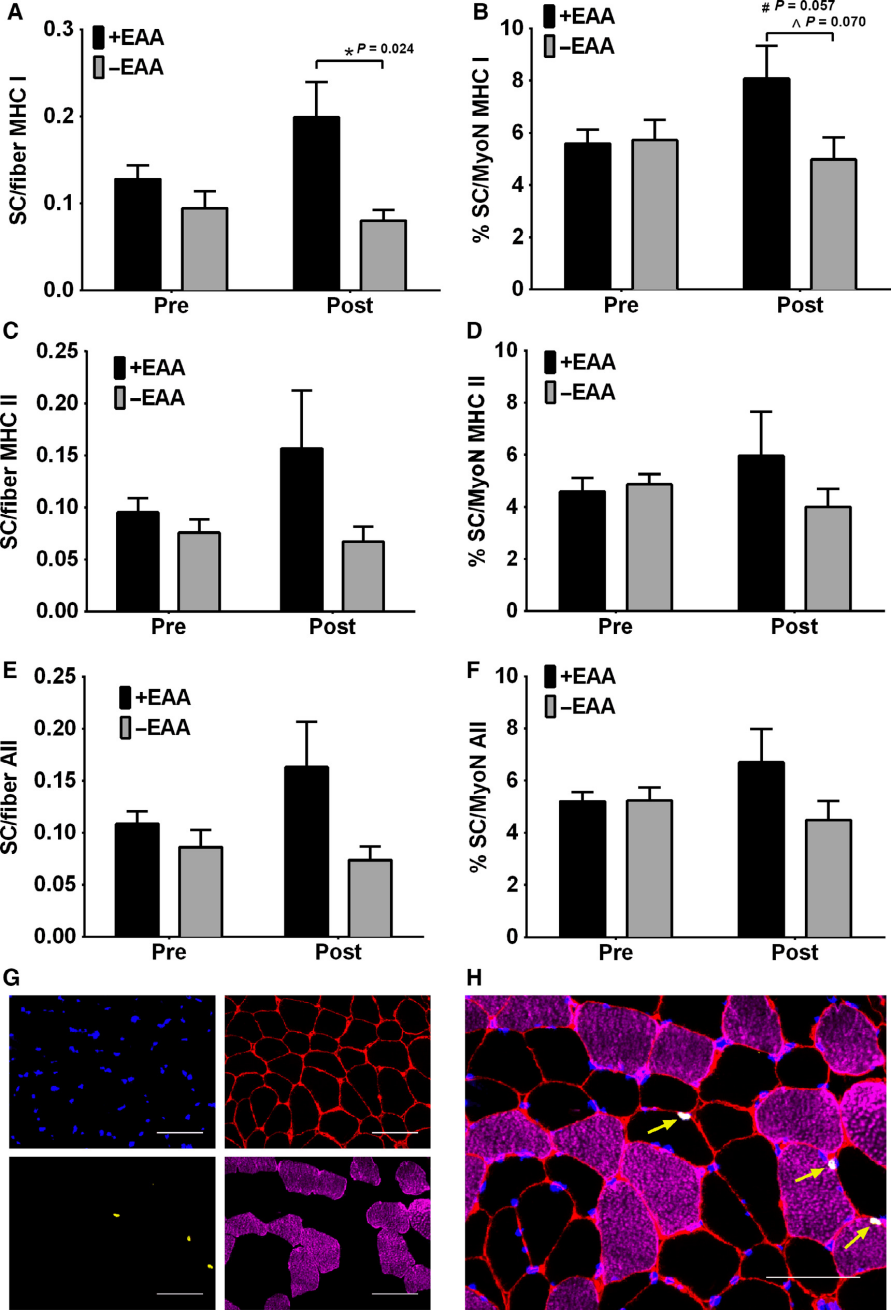
Fiber‐type specific satellite cell content with EAA ingestion postexercise in older men. Skeletal muscle *vastus lateralis* Pax7 +  satellite cell content per myofiber (A,C,E) and percent per myonuceli (B,D,F) in older adult men before (Pre) and 24 h (Post) following a bout of high‐intensity resistance knee extension exercise with (+EAA) or without (−EAA) postexercise EAA ingestion in myosin heavy chain (MHC) I (A,B), MHC II (C,D) and All myofiber types pooled (E,F). (G) Representative individual channel images denoting DAPI (blue), laminin (red), Pax7 (yellow) and MHC I (purple). (H) Merged image demonstrating fiber‐type specific Pax7 +  satellite cell content (yellow arrows). Scale bar = 100 *μ*m. Data are mean ± SEM. # *P* < 0.10 versus Pre for that group, * *P* < 0.05 treatment effect at Post, ^ *P* < 0.10 treatment effect at Post.

Pax7 +  satellite cells per myofiber for MHC I, MHC II and all myofiber types pooled were not different at pre (*P* > 0.10, Fig. 3) and there were no main effects of time or interactions (*P* > 0.10). Yet MHC I Pax7 +  satellite cells per myofiber exhibited a main effect of treatment (*P* = 0.04), which was made evident by a significantly greater post value in +EAA versus −EAA (*P* = 0.02). Although there were no main effects of time, exploratory Silak comparisons of pre versus post indicated that +EAA was approaching statistical significance (*P* = 0.104). Although, this same pattern was visually evident for MHC II and all myofiber types pooled, there were no main effects of time, treatment or interactions (*P* > 0.10).

Pax7 +  satellite cells per myonuclei for MHC I, MHC II and all myofiber types pooled were not different at pre (*P* > 0.10) and there were no main effects of time of interactions (*P* > 0.10). However, MHC I Pax7 +  satellite cells per myonuclei exhibited a trend for an interaction (*P* = 0.07), which was made evident by trends for a significantly greater post value in +EAA versus −EAA treatment (*P* = 0.07) and an increase in +EAA at post (*P* = 0.06). Although, this same pattern was also visually evident for MHC II and all myofiber types pooled, there were no main effects of time, treatment or interactions (*P* > 0.10).

### Muscle MyoD+ cells

**Figure 4:**
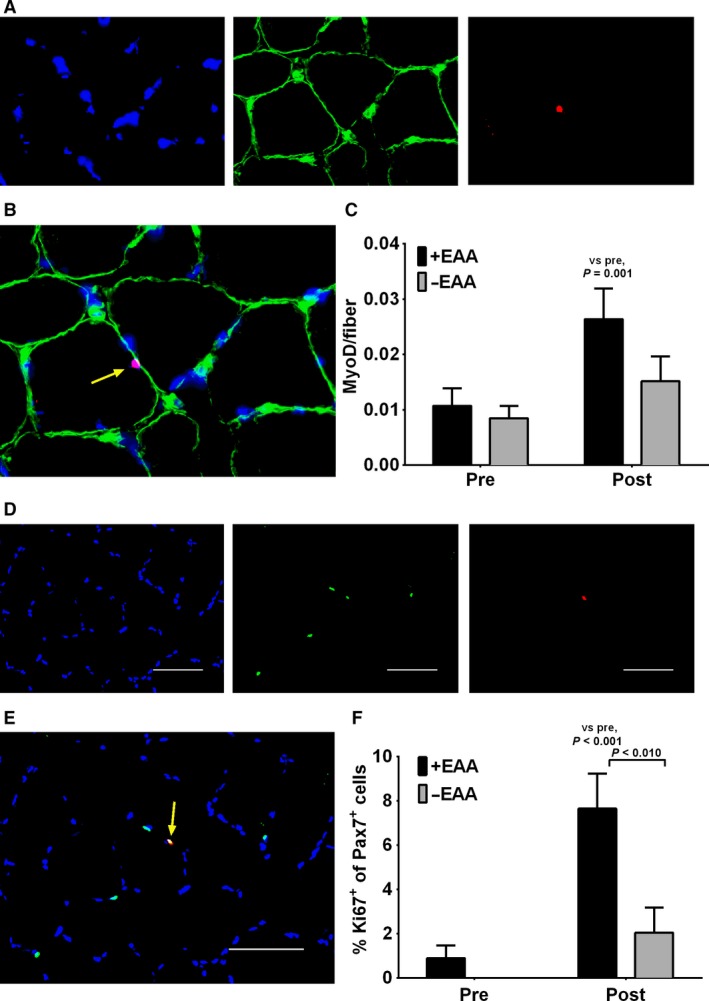
Increased satellite cell proliferation and activation following EAA ingestion in older men postresistance exercise. (A) Representative individual channel images demonstrating DAPI (blue), laminin (green) and MyoD (red) in skeletal muscle *vastus lateralis* in older adult men. (B) Merged image demonstrating a MyoD+ satellite cell (yellow arrow). Scale bar = 100 *μ*m. (C) MyoD+ satellite cell content before (Pre) and 24 h (Post) following a bout of high‐intensity resistance knee extension exercise with (+EAA) or without (−EAA) post‐exercise. (D) Representative individual channel images demonstrating DAPI (blue), Pax7 (green) and Ki67 (red) in skeletal muscle biopsies from older men. (E) Merged image demonstrating a Ki67 +  satellite cell (yellow arrow). Scale bar = 100 *μ*m. (F) Frequency of Ki67 +  satellite cells before (Pre) and 24 h (Post) following a bout of high‐intensity resistance knee extension exercise with (+EAA) or without (−EAA) postexercise. Data are mean ± SEM.

MyoD+ cells per myofiber were not different at pre (*P* > 0.10, Fig. 4A–C) and there were no main effects of treatment or interactions (*P* > 0.10). MyoD+ cells per myofiber exhibited a main effect of time (*P* < 0.01), which was made evident by a significant increase in only the +EAA treatment (*P* < 0.01).

### Pax7 + /Ki67 +  Proliferating Satellite Cells

Pax7 + /Ki67 +  proliferative satellite cells were not detectable in −EAA at pre and only a small proportion was found in +EAA different at pre. There were main effects of time, treatment and an interaction (*P* < 0.05, Fig. 4D–F), which was made evident by a significant increase in +EAA, but not −EAA, from pre to post (*P* < 0.01) and a greater post value in +EAA versus −EAA treatment (*P* < 0.01).

### Change in satellite cell content

**Figure 5:**
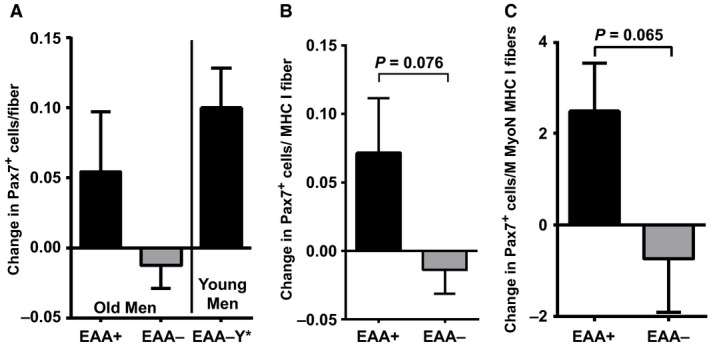
Change in satellite cell content with EAA ingestion 24 h postexercise in older men versus no EAA ingestion in old men and previously published reference data in young men. Skeletal muscle *vastus lateralis* Pax7^+^ satellite cell content per myofiber change from (Pre) to 24 h (Post) following a bout of high‐intensity resistance knee extension exercise with (+EAA) or without (−EAA) postexercise EAA ingestion in older adult men and in comparison to previously published (Walker et al. 2012) reference data in young men (‐EAA‐Y). (A) Change of Pax7^+^ cells in myosin heavy chain (MHC) I myofibers expressed as #/myofiber (B) and percent per myonuclei (C). Data are mean ± SEM.

Pax7 +  satellite cells exhibited an increase in +EAA and a slight, nonsignificant (*P* > 0.10) decrease in −EAA (Fig. 5). Satellite cells associated with MHC Type I fibers, presented both per fiber and per myonucleus, demonstrated a trend for a difference in −EAA versus +EAA when looking at the change in satellite cells pre to post (*P* = 0.076 and 0.065, respectively).

## Discussion

We demonstrate that older men do not appear to increase skeletal muscle satellite cell content at 24 h following heavy, high volume resistance exercise in the absence of EAA ingestion. However, when 10 g of EAA is ingested one hour postexercise we found that MHC I myofiber satellite cell content displays obvious trends to be greater than when older men are not given postexercise EAA. Although this pattern is visually evident in MHC II myofibers and when all myofibers are pooled, the current data set did not reach statistical significance. We compared the pooled data (Pax7 +  cells/fiber) to a previously published Pax7 response in young men undergoing an identical study design in our laboratory (Walker et al. 2012) and discovered that although the response was not restored to the same capacity as younger men, the EAA ingestion in older men promoted partial restoration of Pax7 content.

In support of satellite cell proliferation that occurred following +EAA treatment, we observed increased mRNA expression of CDK2, RB1, NPM1, MDM2, and MEF2C in +EAA at 24 h postexercise. Particularly interesting is the fact that mTORC1 complex activity likely mediates its effects on satellite cell differentiation through various cellular factors such as NPM1, MDM2 and RB1 and MEF2C. Nucleophosmin (NPM1) promotes proliferation, but not differentiation (Qing et al. 2008). Retinoblastoma 1 (RB1) is required for progression through myogenic differentiation but not maintenance of terminal differentiation (Huh et al. 2004). Myocyte‐specific enhancer factor 2C (MEF2C), is a transcription factor in the Mef2 family required for adult muscle regeneration through promotion of differentiation (Liu et al. 2014). Furthermore, NPM1, RB1, and MEF2C were elevated in EAA+ and significantly greater than EAA‐ at 24 h postexercise suggesting enhanced satellite cell proliferation following EAA ingestion in older men. Mouse double minute 2 homolog (MDM2) is a negative regulator of p53 and also acts as an E3 ligase (Signer Robert and Morrison Sean 2013). MDM2 may serve to protect satellite cells from ROS‐induced apoptosis triggered through p53 (Abbas et al. 2010; Signer Robert and Morrison Sean 2013; Pant et al. 2013) and MDM2 is necessary for myogenic differentiation (Fu et al. 2015). Interestingly, with the exception of MDM2, all these genes encode proteins that function to promote differentiation but not necessarily differentiation in muscle. This presents the possibility that the proliferating satellite cell pool is functioning to some other unknown fate in muscle (maintenance of the myofiber environment, etc.) in lieu of the traditional role of myonuclear commitment. Since many of these mRNA species are not exclusive to satellite cells this interpretation of whole muscle mRNA data is speculative, yet we feel supported by our IHC data showing satellite cell proliferation.

It is well understood that resistance exercise increases the satellite cell pool in younger adults (McKay et al. 2012, 2013; Snijders et al. 2014), as we have demonstrated previously (Walker et al. 2012). Further, our laboratory has previously demonstrated a blunting of the postexercise satellite cell pool in older adults (Walker et al. 2012), which has also been demonstrated for eccentric exercise (Dreyer et al. 2006) and also in a fiber‐type specific manner (McKay et al. 2012, 2013; Snijders et al. 2014). These data suggest that older adults have a blunted or delayed ability to expand the satellite cell pool in response to an acute about of exercise, presumably due to greater stress/inflammation (McKay et al. 2013). Yet it appears that as older adults adapt to resistance exercise training they eventually expand the satellite cell pool in skeletal muscle in some cases (Roth et al. 2001; Mackey et al. 2007; Verney et al. 2008; Verdijk et al. 2009a), but not all (Petrella et al. 2006). Interestingly, recent work from Farup et al. (2014) supports our data by demonstrating that whey protein ingestion accelerates the satellite cell pool expansion at 48 h following eccentric RE in young adults. However, they showed that after ~1 week following the commencement of the exercise, the satellite cell pool was identical between treatments, suggesting that amino acid provision postexercise merely altered the time course of satellite cell proliferation. We are the first to demonstrate that older men may also benefit from postexercise ingestion of amino acids in a similar regard.

EAA ingestion in older men promoted partially restored Pax7 content at 24 h post‐RE compared to a previously published Pax7 response in young men (Walker et al. 2012). Although, we did not see a statistical increase in the +EAA group, we demonstrated a significant difference between groups postexercise and a clear trend in MHC I myofibers. Indeed, 24 h post‐RE may have been too early to detect a significant effect of EAA since older men do not start to increase satellite cell content until upwards of 48–72 h post‐RE (Snijders et al. 2014). Although, this study lacks sufficient sample size to detect significant increases in Pax7^+^  cell content, as early as 24 h post‐RE, it is important to note that, these data were further supported by clear evidence of enhanced satellite cell proliferation through two separate indices (Ki67^+^  and MyoD^+^ cells) at 24 h post‐RE in older men. This is important to note because older men have a delayed and diminished ability to increase MyoD^+^/Pax7^+^ cells compared to young men, who increase the number of these proliferative cells as early as 12 h post‐RE (Snijders et al. 2014). The lack of an effect in MHC II myofibers, in older adults is in agreement with a previous report (McKay et al. 2013), however, similar to the MHC I response it is possible that an effect of EAA could be observed at a later time point. Based in the findings by Farup et al. (2014) it is unlikely that protein or amino acid supplementation will enhance the satellite cell pool following resistance exercise training (RET), at least for eccentric exercise. Indeed, it appears that older adults do not have an added effect of protein supplementation (Verdijk et al. 2009a, 2009b; Molsted et al. 2015) on the satellite cell pool following chronic RET. Thus, it is likely that an effect of EAA supplementation on the satellite cell pool would be limited to the first few days into the start of exercise training. Although the exact role of this enhanced satellite cell pool is unknown to us, we conclude that it is likely (during this time of muscle remodeling) these satellite cells will contribute towards maintenance of the myofiber environment (Fry et al. 2014a, 2015) to facilitate regeneration following myofiber damage from exercise in aging muscle. Further, a study has shown that leucine supplementation 3 days following muscle injury increases MyoD^+^Pax7^+^ cells (Pereira et al. 2015), which provides support to this hypothesis that amino acids can enhance the proliferative action of satellite cells to promote muscle regeneration following muscle damage.

## Conflict of Interest

P.T. Reidy, C.S. Fry, J.M. Dickinson, M.J. Drummond, and B.B. Rasmussen have no conflicts of interest.

## Data Accessibility

## Supporting information




**Table S1.** Primer sequences and Assay ID used for real‐time PCR.
