# Supplementary material for: Postexercise essential amino acid supplementation amplifies skeletal muscle satellite cell proliferation in older men 24 hours postexercise
Source: Physiol Rep. 2017 Jun 8;5(11):e13269. doi: 10.14814/phy2.13269 (PMC5471431; doi:10.14814/phy2.13269)
Supplement: Supplementary file 2 [file PHY2-5-e13269-s002.docx]

**Table S1. Primer sequences and Assay ID used for real-time PCR**
